# The Effect of Various Grinding Aids on the Properties of Cement and Its Compatibility with Acrylate-Based Superplasticizer

**DOI:** 10.3390/ma15020614

**Published:** 2022-01-14

**Authors:** Ewa Kapeluszna, Łukasz Kotwica

**Affiliations:** Faculty of Materials Science and Ceramics, AGH University of Science and Technology, 30-059 Krakow, Poland; lkotwica@agh.edu.pl

**Keywords:** grinding aids, acrylate superplasticizer, viscosity, rheology, triethanolamine, ethylene glycol

## Abstract

The influence of grinding aids (pure triethanolamine and ethylene glycol) on the properties of cements, their compatibility with an acrylate-based superplasticizer and the rheological parameters of mortars were investigated. The presence of surfactants influences the standard properties of cements and the effectiveness of the superplasticizer. The results of the heat of hydration and setting time measurements indicate a delay in the hydration process and an increase in the induction period duration of the surfactant-doped pastes, in relation to the reference sample without grinding aids. Triethanolamine increases early-age compressive strength; the effect was observed for both standard and superplasticizer-containing mortars. The presence of grinding aids decreases the slump flow of mortars and increases rheological parameters such as yield stress (*τ*_0_) and viscosity (*η*).

## 1. Introduction

Grinding aids, first introduced into cement manufacturing in 1930 [1], are surface-active substances that facilitate particle comminution during the milling process. In the production of Portland cement, grinding aids are added in small amounts in the range of 0.01 to 0.10% by weight of cement, according to the PN-EN 197-1 standard [1]. They allow a larger specific surface area to be obtained by the material in a shorter grinding time, which reduces energy consumption. The literature shows that even such a small addition of these agents can increase the efficiency of the mills by 15 to 25% [2]. The reduction in grinding costs should be greater than the price of the agent.

Numerous organic and inorganic substances, as well as mixtures thereof, are used as grinding aids in the production of Portland cements. In terms of chemical composition, they can be divided into amines and their salts, polyalcohols, lignosulfonates, fatty acids and fatty acid salts [3]. The most effective grinding aids are surfactants, including propylene glycol, triethanolamine, triethanolamine acetate and polyglycol phenol ether [1,2,3].

The grinding of Portland clinker leads to the formation of new microcracks in the crystal structure. When the defects are formed, the ionic bonds in the crystals are broken, and the grains gain high surface energy. As a result of this phenomenon, positive and negative charges arise on the surface of cement grains, which cause them to interact with each other, leading to unfavorable agglomeration. The general effect of surfactants is related to their interaction with the surface of the anhydrous cementitious phases—they form layers that prevent the grains from ‘sticking’ to each other. Their mechanism of action is based on the reduction of the material’s resistance to fragmentation and the prevention of agglomeration [1]. During the comminution process, the mechanical stresses act discontinuously, and in the inactive period, the microcracks may be sealed. The surfactants adsorb inside these microcracks, which in turn hinders their resealing and prevents the agglomeration of the grains.

There are two main mechanisms that explain the dispersion of surfactants: transfer through the gas phase and transfer through surface contact. The temperature in the mill is usually in the range of 80–120 °C, and the adsorption capacity of the compounds is strongly dependent on their boiling point. Most of the surfactants used (e.g., polyglycol—PG, triethanolamine—TEA, ethylene glycol) have a boiling point above the milling temperature and therefore, they are dispersed by both mechanisms [4,5].

The application of surfactants as grinding aids changes certain cement properties, such as water demand, setting times and compressive strength [1,2,6,7]. Grzymek et al. [8,9] investigated the effect of waste ethylene glycol addition and reported that it increases the water demand and setting time of cement. Heren studied the impact of surfactants based on ethylamine (MEA—monoethyleneamine, DEA—diethyleneamine, TEA—triethyleneamine). The results showed a delay in hydration and an increase in the compressive strength [10]. Katsioti confirmed the beneficial effect of amines (triisopropanolamine—TIPA) on strength; however, the setting time of cement was extended [11]. Aiad et al. presented the effect of amines on the rheological parameters of mortars. TEA decreased the viscosity. Explanations of the chemical action mechanism of these additives have also been presented in the literature. The theory is based on the phenomenon of delayed hydration of C_3_S and β-C_2_S in the presence of TEA, resulting in a longer induction period [12,13,14,15]. An important finding is that the C_3_A reaction accelerates in the presence of TEA [16,17,18]. The latest research on triethanolamine shows that it decreases the content of Ca(OH)_2_ and changes the microstructure of cement mortars. The conclusions were supported by transmission electron microscopy observations, as well as by the determination of the calcium hydroxide content by differential thermal analysis. The phenomenon was explained by the formation of complex compounds with Ca^2+^ [19,20,21]. Assaad and Issa compared the effect of grinding aids based on triethanolamine (TEA) and glycol (GLI) in different variants [7]. They investigated the impact of these additives on variations in the flow of cement pastes, including static yield stress and viscosity. Milling cements with additional grinding aids resulted in a reduced setting time and an improved compressive strength. This was attributed to a physical effect related to a higher packing density and increased formation rates of early hydrates that create additional interparticle links in the suspension.

Due to the significant progress in the production of new types of superplasticizers, the problem of their compatibility with cement is still current. In the literature, various explanations can be found on the reasons for the incompatibility of water-reducing admixtures with cement [22,23,24,25,26,27,28,29]. Polycarboxylate superplasticizers show different efficiencies, depending on the w/c ratio, the C_3_A phase content, as well as the amount of sulfates introduced into the cement. The increase in sulfates content reduces the adsorption of the plasticizer and decreases the fluidity [22,23,24,25]. Accordingly, the increase in the aluminate content has to be compensated by the increase in the sulfate content. The time at which the admixture is introduced into the mortar or concrete mix is also of great importance. It is recommended to add the superplasticizer by the end of the mixing process, in order to avoid the superplasticizer making contact with dry cement surface [25,26]. The use of various types of mineral additives also influences the action of superplasticizers.

Multivariate analysis leads to the conclusion that each concrete and mortar recipe should be considered separately in terms of selecting the type and amount of superplasticizer used [28,29,30]. A very important issue in the field of superplasticizer development is the compatibility with admixtures that accelerate the hardening process—TEA, calcium nitrates and nitrites (CNN), sodium aluminate (SA), etc. In their work [30], the authors report that triethylamine used in the binding materials industry as a setting accelerator, when dosed in an amount above 0.4%, increases the C_3_A hydration rate by altering the reaction between C_3_A and gypsum [31].

Cementitious pastes are thixotropic, non-Newtonian liquids, for which the flow curve (a function of shear rate in a rotational rheometer) is not constant over time; therefore, they show variations in viscosity and flow limit. This article presents the results of research on the influence of surfactants, such as ethylene glycol and triethanolamine, on the properties of cements, as well as on the rheological parameters during the early hydration period. Moreover, the compatibility of triethanolamine and ethylene glycol with the acrylate-based superplasticizer was investigated by determining the changes in the aforementioned properties.

## 2. Materials and Methods

Three cements were ground in a laboratory ball mill to a specific surface area of 4200 cm^2^/g (according to Blaine). Cement composition was constant for all investigated samples: 95% of Portland clinker and 5% of anhydrite (by mass). Such approach allowed the influence of each grinding aid on the properties of the obtained binder to be investigated. Table 1 shows the chemical and phase compositions of cement, determined by XRF (Panalytical WDXRF Axios mAX with Rh lamp, Malvern, UK) and Bogue’s formulas with Taylor’s corrections [2]. Table 2 shows the mix proportions of raw materials subjected to grinding. The reference cement (CEM ‘0’) was milled without any grinding aids. The other two were ground with triethanolamine (TEA) and ethylene glycol (GLY). During milling, the specific surface area was controlled with a Blaine apparatus (Acmel labo BSA-1, Saint-Pierre-du-Perray, France). The final grain size distribution of the binders was determined with a laser diffractometer (Malvern Panalytical Mastersizer 2000, Malvern, UK), using isopropyl alcohol as a carrier.

The heat of hydration evolution was measured using a differential heat-conduction microcalorimeter. The reference temperature was maintained at 25 °C. Paste samples were composed of 20 g of cements and 10 g of water (w/c = 0.5).

The consistency of mortars was investigated with a flow table, according to PN-EN 1015-3 [32]. The water demand and setting time were determined in accordance with PN-EN 196-3 [33]. The strength of cement mortars was investigated on 25 × 25 × 100 mm^3^ bar samples with a hydraulic press after 1, 7, 28 and 91 days of curing, according to PN-EN 196-1 [34].

In addition, the rheological properties of cement pastes were tested using an Ofite 900 rotary viscometer with a maximum shear rate of 1022 s^−1^ (600 rpm). The minimum volume of the sample was approx. 300 cm^3^. A w/c ratio of 0.6 was chosen to ensure the proper fluidity of the pastes, fulfilling the demands of the apparatus. It allowed the changes in yield stress (*τ*_0_) and plastic viscosity (*η*) that could not be measured at a low w/c to be traced. The selected type of rheometer eliminates the influence of grain segregation on the measurements, occurring at high water to cement ratios. The procedure is mainly used for well cementing materials (acc. to PN-EN ISO 10426-1:2009 [35]).

The compatibility of the system: cement—grinding aids—acrylate-based superplasticizer was also investigated. Two types of standard mortars—with and without a superplasticizer (SP)—were prepared for consistency and strength evaluations. Their cement content was increased to prevent segregation and to better emphasize the physicochemical phenomena occurring during hydration. Moreover, the w/c ratio was lowered from 0.5 to 0.4 when SP was used. Furthermore, rheological parameters were tested on pastes prepared at a w/c ratio of 0.4 (with SP) and 0.6 (without SP). The compositions of mortars and pastes are presented in Table 3 and Table 4.

## 3. Results

### 3.1. Grindability and Grain Size Distribution of Cements

**Figure 1:**
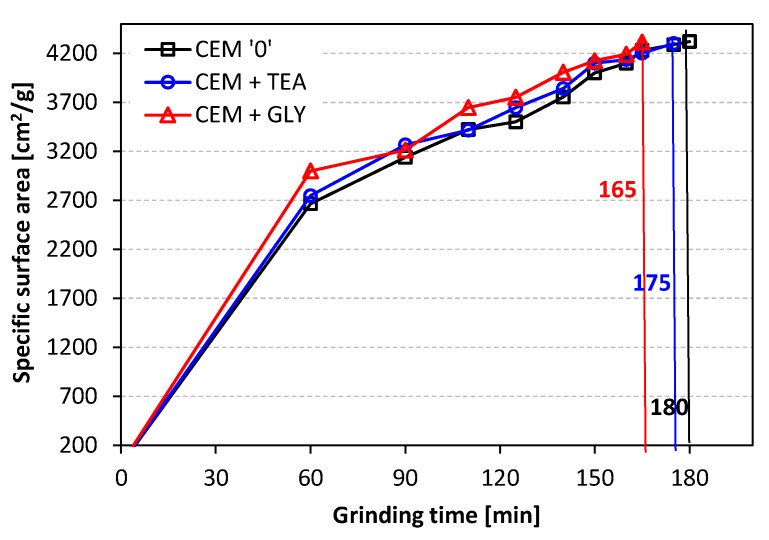
Specific surface area of cements vs. grinding time.

To compare the efficiency of the grinding process with and without grinding aids, changes in the specific surface area of the cements as a function of grinding time were investigated (Figure 1). First, all cements were ground for 60 min, after which their surface area was determined using a Blaine apparatus. Cements were then further ground, and their specific surface area was measured at shorter time intervals. Details are shown in Figure 1. The total cement grinding time required to obtain a specific surface area of 4200 cm^2^/g was the shortest for ethylene glycol (165 min), indicating a better efficiency of this additive. The grinding time of cement with triethanolamine was 175 min. The reference cement had to be ground for 180 min.

**Figure 2:**
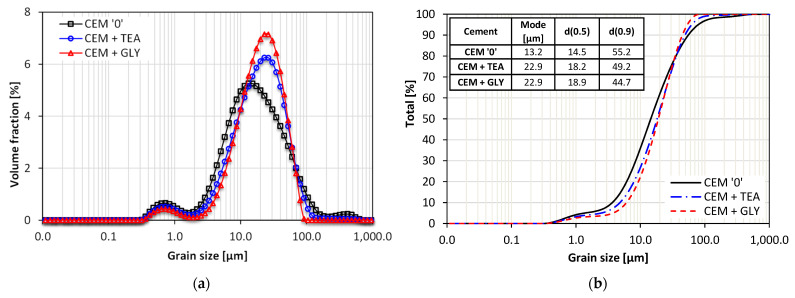
Grain size distribution of cements: (**a**) differential and (**b**) cumulative curves.

The grain size distribution of cements with grinding aids is narrowed, compared with the reference cement without additives, which means that size of their grains is more homogeneous (Figure 2). Slight differences can be noticed in the range of 10 ÷ 40 µm, where CEM ‘0’ has about 30% fewer grains, compared with CEM + TEA and CEM + GLY. However, the control cement contains more fine grains, within the range of 2 ÷ 10 µm. It can be said that the grain size distribution of CEM + TEA and CEM + GLY cements is slightly shifted towards smaller grain sizes.

### 3.2. Standard Properties of Cements

**Figure 3:**
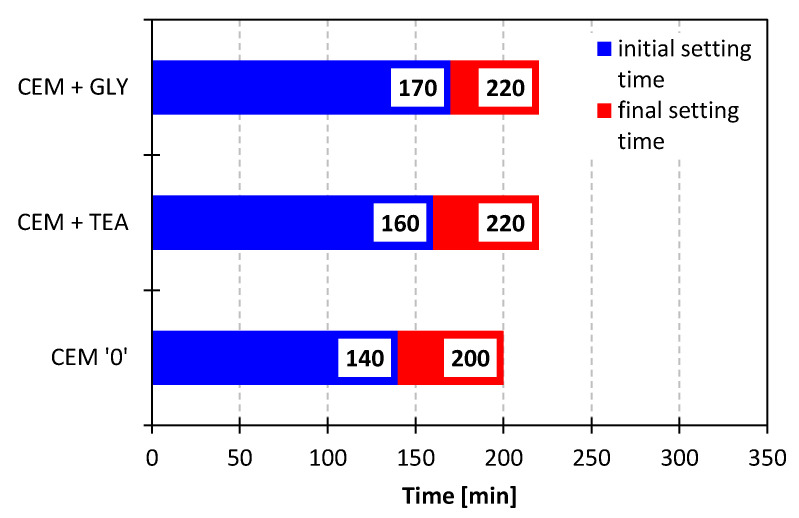
Initial and final setting times of cements.

As shown in Table 5 and Figure 3, cements obtained with the use of admixtures are characterized by an increased setting time, compared with the reference binder. The initial setting time was 30 min longer for glycol and 20 min longer for triethanolamine. This outcome is most likely related to the higher water demand of cements ground with the grinding aids (which increased by approx. 2.0–2.4 percentage points). Moreover, as described in the literature, triethanolamine delays the hydration of C_3_S, the phase responsible for the initial strength of the cement-based composites [16,17].

**Figure 4:**
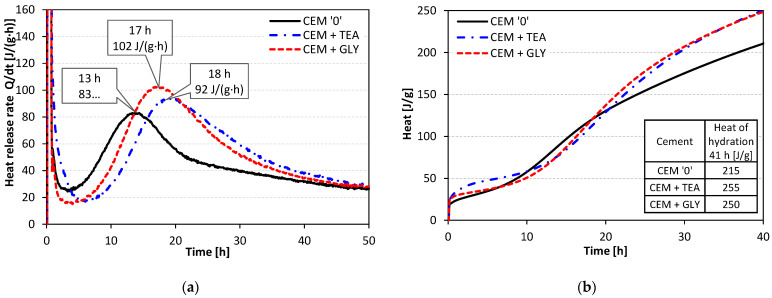
Heat evolution curves of cements: (**a**) heat release rate, (**b**) cumulative heat.

The analysis of heat evolved during hydration (Figure 4a) allows for the conclusion that surface-active substances influence the hydration rate despite their very small mass share (0.1%). This is evidenced by significant differences in the course of the microcalorimetric curves. The induction period of cements containing TEA and GLY is slightly longer than that of the reference. Therefore, they are characterized by a slightly lower heat evolution rate in the initial period, up to approx. 12 h of hydration. After this time, the heat evolution rate is higher in the case of cements with grinding aids. Consequently, the total heat released after 41 h is approx. 20% higher for cements with TEA and GLY, compared with CEM ‘0’ (Figure 4b). It should be emphasized that the results of calorimetric measurements correlate well with the setting times.

**Figure 5:**
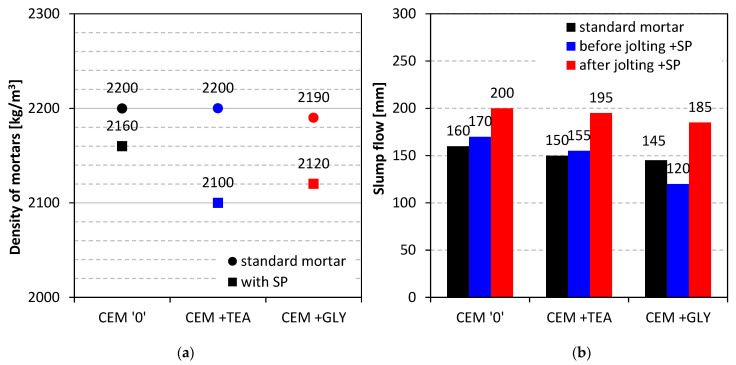
(**a**) Density and (**b**) slump flow of mortars with and without superplasticizer.

The consistency of fresh mortars was determined in two ways to evaluate both the impact of grinding aids on mortar workability and their compatibility with an acrylate-based superplasticizer. The slump flow of mortars with SP was measured before and after the table was jolted (according to PN-EN 1015-3). The results are presented in Figure 5b. Even in the case of standard mortars, the differences in the consistency of the mixture are visible. Cements containing glycol and triethanolamine show a 10 and 15 mm lower slump flow, compared to the reference mortar. This outcome can be explained by the more homogenous grain size distribution of those cements, as well as by the increase in the viscosity of the mortar due to the presence of surface-active grinding aids. In the case of mortars with SP, more significant changes were observed. Before the table was jolted, the initial slump flow of CEM + TEA and CEM + GLY mortars was 9% and 30% lower, respectively, compared with the reference sample. The situation changed after the jolting. The differences in flow were much slighter for CEM + TEA and CEM + GLY, the values were about 5 and 15 mm lower than the reference. The effectiveness of the superplasticizer was the lowest for the glycol-containing cement, compared to both the reference and the TEA-containing cement. The consistency results gave the basis for further rheological studies and considerations on the compatibility of the two organic admixtures used as grinding aids (TEA or GLY) and acrylic-based superplasticizer, described in Section 3.3 of the paper.

**Figure 6:**
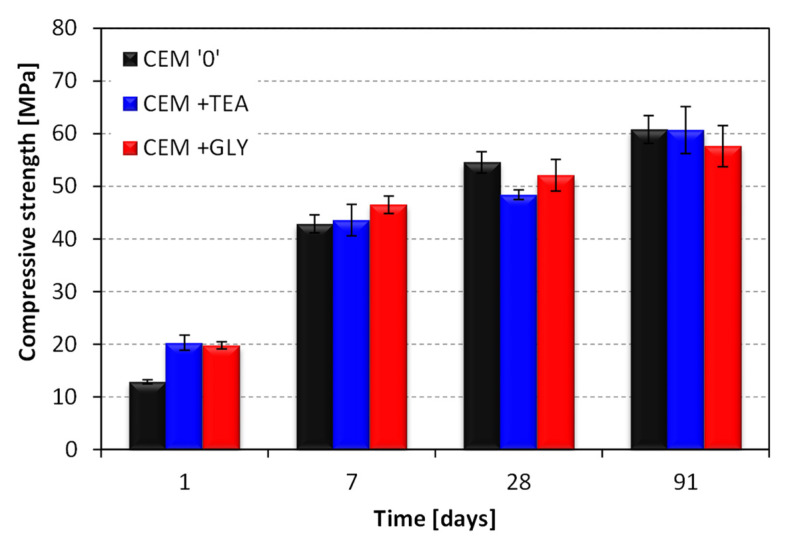
Compressive strength of standard mortars, w/c = 0.5.

The mortar prepared with the reference cement was characterized by a lower compressive strength after 1 day of hydration, compared with the mortars containing cements ground with surfactants (Figure 6). The results evened out after longer curing periods. After 7 days of hydration, the strength was similar in all series, whereas after 28 days, it was 11% and 5% lower for CEM + TEA and CEM + GLY mortars, respectively, compared to the reference sample. The influence of ethylene glycol and triethanolamine on the mechanical performance of mortars correlates well with the already published data [7].

**Figure 7:**
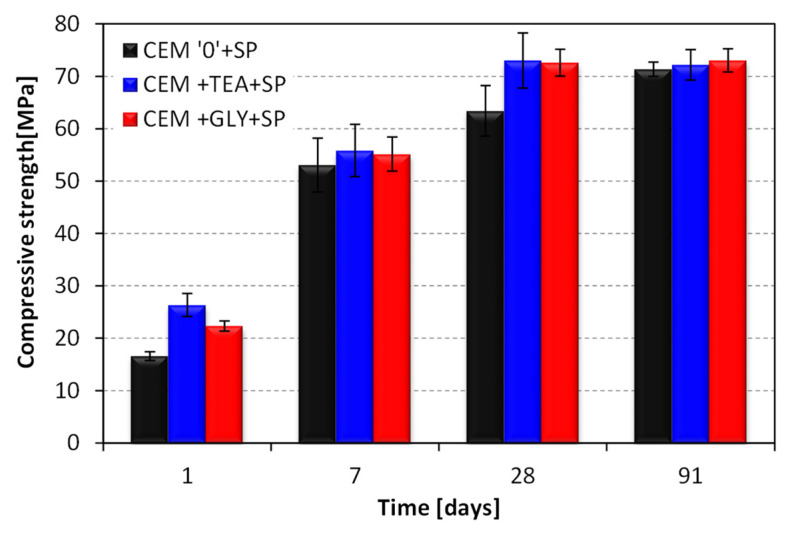
Compressive strength of mortars with superplasticizer, w/c = 0.4.

In the early stage of curing (1 day), the test results for the composites with acrylic-based superplasticizer correlate with those obtained for standard mortars (Figure 7). The compressive strength of samples containing cements ground with TEA and GLY was approximately 58% and 34% higher than the reference. It can be concluded that the introduction of triethanolamine and ethylene glycol during grinding has a positive effect on the early (1 day) compressive strength of mortars, both with and without the superplasticizer. After longer curing periods, the grinding aids have a more beneficial effect in the case of superplasticized mortars. On the 90th day, the strength of mortars with grinding aids was close to that of the corresponding reference mortar.

### 3.3. Rheological Properties of Cement Pastes: Viscosity η and Yield Stress τ_0_

Compared to standard workability measurements, the evaluation of rheological properties allows for a more fundamental investigation and a more precise description of flow properties. Therefore, it is a key in explaining the observations made during the standard research on mortars and pastes, conducted in the early hydration period. The most common method used to determine the rheological properties of pastes is to record their flow curves. On the basis of suitable models, rheological parameters can be calculated. Such parameters are used not only in the qualitative analysis of the observed phenomena, but also in other calculations, for example, in the drilling industry, where the resistance to flow is of key importance [36].

**Figure 8:**
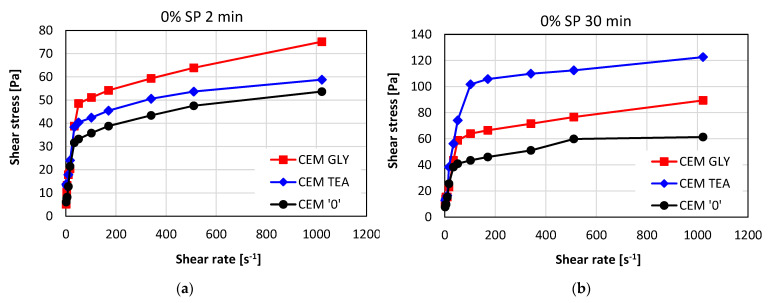
Flow curves of cement pastes without superplasticizer after (**a**) 2 min and (**b**) 30 min of hydration; w/c ratio = 0.6.

**Figure 9:**
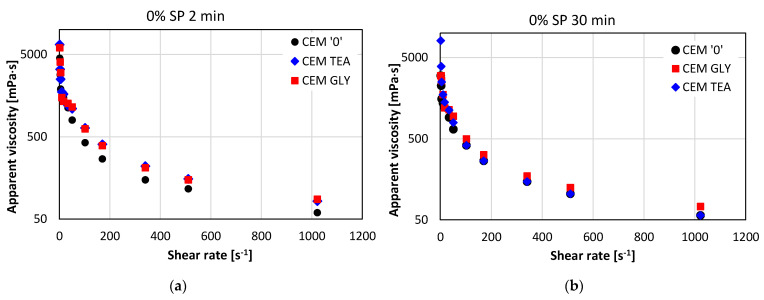
Apparent viscosity of cement pastes without superplasticizer after (**a**) 2 min and (**b**) 30 min of hydration.

Figure 8 presents the flow curves for pastes without superplasticizer, collected after two different cement hydration times: immediately after mixing with water (2 min—Figure 8a) and after 30 min (Figure 8b). Substantial differences can be seen between the reference cement and the cements with grinding aids. As is visible, the curves are nonlinear. The apparent viscosity of all the pastes is decreasing with the increasing shear rate, which means that all the pastes exhibit shear thinning. Figure 9 shows how the apparent viscosity of the pastes changes with the increasing shear rate.

In order to quantify the differences in the rheology of particular pastes, we decided to involve rheological models. Numerical calculations, made using Rheosolution software (the Rheosolution software is property of the Department of Drilling and Geoengineering, AGH University of Science and Technology [37], allowed the analysis of the best rheological models among those most commonly used (Newton, Bingham, Casson, Ostwald de Waele, Hershel-Bulkley), fitting the actual data obtained during flow curve determination. It was found that the obtained curves should be analyzed separately for the low-range shear rate (up to 30 s^−1^) and the high-range shear rate (100–1022 s^−1^). The Bingham model was used, described by the following equation:

(1) $$\tau =\tau_{0}+\eta_{p}\;\dot{\gamma }$$

where:

τ—shear stress, Pa;
$\tau_{0}$—yield stress, Pa;
$\dot{\gamma }$—shear rate, s^−1^;
$\eta_{p}$—plastic viscosity, Pa∙s.

**Figure 10:**
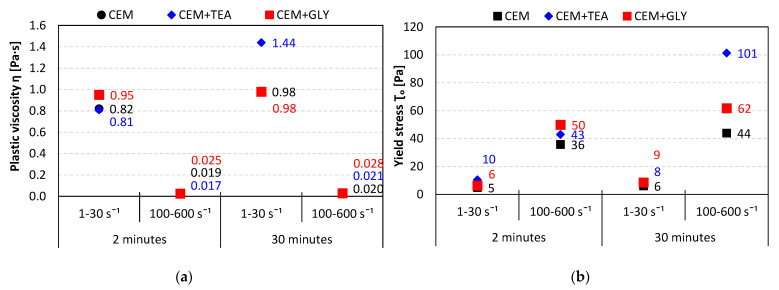
(**a**) Plastic viscosity and (**b**) yield stress of pastes without superplasticizer; w/c = 0.6.

In those two ranges, a good fitting was obtained. In most cases, the Pearson correlation coefficient was greater than 0.97. On the basis of the parameters obtained from the regression equations, rheological parameters: yield stress (*τ*_0_) and plastic viscosity (*η*) were determined for both ranges. Their values are presented in Figure 10a (*η*) and Figure 10b (*τ*_0_).

Depending on the shear rate range, different regularities can be observed. The viscosity is much higher for low shear rates than it is for high shear rates. The presence of grinding aids results in an increase in plastic viscosity at low shear rates, while at high shear rates, their impact is not as pronounced. In general, pastes made of cement containing grinding aids have a worse fluidity, compared to neat cement. The differences become more significant after 30 min of hydration. This may be explained by a higher rate of hydration in the initial stage (see Figure 4). The heat evolution of CEM + TEA was more pronounced compared to other pastes, which corresponds to the changes in yield stress changes after 30 min of hydration. Plastic viscosity at low shear rates was also markedly increased in the presence of TEA, suggesting a faster formation of hydration products, especially ettringite. No XRD investigations were performed in order to confirm this hypothesis, however, works of Ramachandran et al. [17] showed that tricalcium aluminate hydration is accelerated in the presence of TEA.

**Figure 11:**
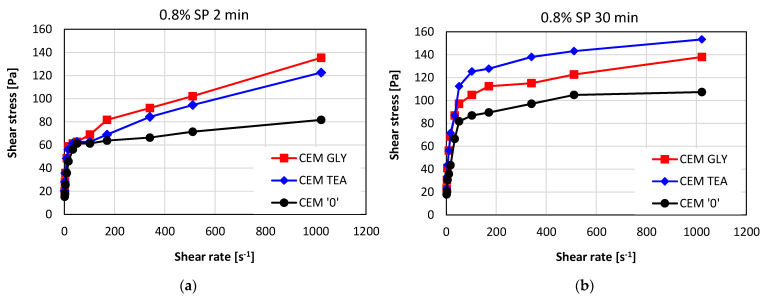
Flow curves of cement pastes containing an acrylic-based superplasticizer after (**a**) 2 min and after (**b**) 30 min of hydration; w/c = 0.4.

**Figure 12:**
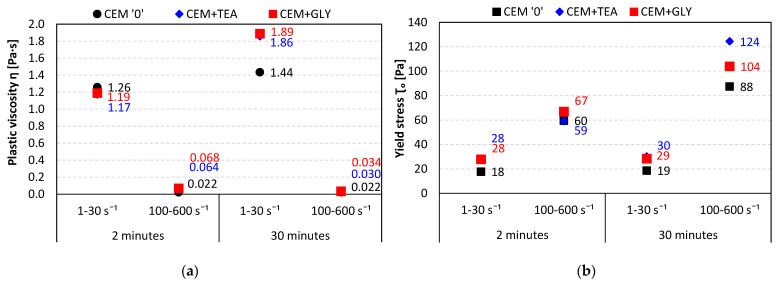
(**a**) Plastic viscosity and (**b**) yield stress of pastes containing an acrylic-based superplasticizer, w/c = 0.4.

Flow curves were also measured for pastes containing 0.8% of superplasticizer in respect to the mass of cement, characterized by a lowered w/c ratio, equal to 0.4. Figure 11a,b present the results obtained after 2 and 30 min of hydration. In Figure 12, the plastic viscosity (*η*—Figure 12a) and yield stress (*τ*_0_—Figure 12b) values are compared. The presence of grinding aids influences the rheological properties especially after 30 min of hydration. Both the yield stress and viscosity of the superplasticized pastes are higher, compared with the control paste without grinding aids. This corresponds well with the slump flow results obtained for mortars. The values obtained for CEM GLY and CEM TEA mortars were lower, compared with neat cement, which is related to their higher shear stress (see Figure 5b).

The problem of mutual interactions between grinding aids and superplasticizers is not yet well researched. Surely, the factor which influences the efficiency of the superplasticizer is the adsorption of grinding aids on the surface of cement particles. Sun et al. [38] showed that the decrease in surface energy, caused by the adhesion of glycerin used as a grinding aid, reduces the adsorption capacity of a PCE superplasticizer. Further work is needed in order to explain the changes in rheology of the pastes concerning the adsorption degree of TEA and GLY.

Comparison of the influence of grinding aids on the action of a superplasticizer is quite complex. As mentioned above, cements containing grinding aids are less susceptible to fluidization, compared with neat cement.

**Figure 13:**
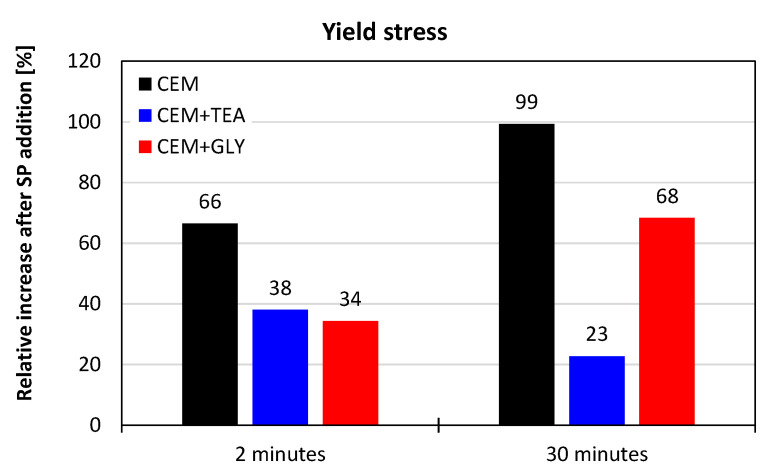
Influence of TEA and GLY on the effectiveness of the superplasticizer—relative increase in the yield stress value after the addition of superplasticizer; data for high shear rates (100–1022 s^−1^).

Considering the relative changes in the yield stress value of the pastes with and without the superplasticizer, it can be concluded that the effectiveness of the admixture is the highest for cement without grinding aids (Figure 13). This means that the presence of triethanolamine and glycol disturbs the positive effect of acrylate-based superplasticizers on the consistency and flowability of OPC pastes.

In general, cements with TEA and GLY perform worse, compared to neat cement, which shows that the subject is complex and requires further research. The topic appears to be of great importance, as the difference in rheology between cements with and without the grinding aids is significant; it is in the range of dozens of percent when rheological properties are taken into account.

## 4. Conclusions

Present research was focused on the influence of two grinding aids—triethanolamine (TEA) and ethylene glycol (GLY)—on the basic properties of cements, cement pastes and mortars. Particular emphasis was put on the rheological properties of pastes with and without a superplasticizer, as this topic is poorly recognized in the literature.

The obtained results show that the incorporation of TEA and GLY improved the grinding efficiency. The time needed to obtain the desired specific surface area of 4200 cm^2^/g was shortened by 8% (GLY) and 3% (TEA). The initial cement setting time was reduced by 10 min and 30 min for GLY and TEA, respectively. The acceleration of hydration in the early period resulted in a greater amount of heat evolved during the reaction. The introduction of ethylene glycol and triethanolamine increased the amount of heat released by 16% and 19%, respectively. A greater amount of heat evolved indicates a higher degree of hydration. As a result, the early compressive strength of mortars containing grinding aids was significantly increased—by up to more than 50% after 1 day. TEA was found to have a more beneficial impact on the early strength development. At later ages, the differences in strength were less significant and finally, after 90 days of hydration, the strength of mortars with grinding aids was similar to that of the control mortar, containing neat cement.

Incorporation of TEA and GLY resulted in a deterioration of pastes rheology, both with and without the superplasticizer. Yield stress and plastic viscosity are generally higher for pastes with grinding aids. However, it seems that despite having the overall worse performance, in terms of rheology, cements with grinding aids react more strongly on the superplasticizer addition, compared with neat cement.

The obtained results highlight the impact of grinding aids on the efficiency and compatibility of superplasticizer with cement. There are relatively large differences between the properties of cements with and without TEA of GLY in terms of fresh mix rheology. This may become a problem for a concrete plant, when the cement manufacturer changes the type or the amount of grinding aid used in the milling process.

## Figures and Tables

**Table 1:** Chemical and phase compositions of cement prepared in a laboratory ball mill.

**Chemical Composition of Cement [%]**								
LOI	CaO	SiO_2_	Al_2_O_3_	Fe_2_O_3_	SO_3_	K_2_O	Na_2_O	Cl
2.08	65.6	22.4	4.3	2.5	3.1	0.5	0.3	0.1
**Phase Composition of Cement [%]**								
C_3_S		C_2_S		C_3_A	C_4_AF		$C\bar{S}$	
55.5		22.7		7.2	7.6		5.3	

**Table 2:** Mix proportions of raw materials subjected to grinding.

Cement	Clinker [%]	Anhydrite [%]	TEA [%]	GLY [%]
CEM ‘0’	95	5	-	-
CEM + TEA	95	5	0.1 *	-
CEM + GLY	95	5	-	0.1 *

* % by mass of cement.

**Table 3:** Composition of mortars for consistency and strength evaluations.

Mortar	Component				w/c
	Cement [g]	Sand [g]	Water [g]	Superplasticizer [%]	
CEM ‘0’ CEM + GLY CEM + TEA	600	1350	300	-	0.5
CEM ‘0’ + SP CEM + GLY + SP CEM + TEA + SP	600	1350	240	0.6	0.4

**Table 4:** Composition of pastes for rheological tests.

Paste	Component			w/c
	Cement [g]	Water [g]	Superplasticizer [%]	
CEM ‘0’ CEM + GLY CEM + TEA	600	360	-	0.6
CEM ‘0’ + SP CEM + GLY + SP CEM + TEA + SP	600	240	0.8	0.4

**Table 5:** Water demand and setting times of cements.

Sample	Water Demand [%]	Initial Setting Time [min]	Final Setting Time [min]
CEM ‘0’	26.4	140	200
CEM + TEA	28.8	160	220
CEM + GLY	28.4	170	220

## Data Availability

The data presented in this study are available on request from the corresponding author.
